# Partial agonist activity of α_1_-adrenergic receptor antagonists for chemokine (C-X-C motif) receptor 4 and atypical chemokine receptor 3

**DOI:** 10.1371/journal.pone.0204041

**Published:** 2018-09-24

**Authors:** Xianlong Gao, Hazem Abdelkarim, Lauren J. Albee, Brian F. Volkman, Vadim Gaponenko, Matthias Majetschak

**Affiliations:** 1 Department of Surgery, Morsani College of Medicine, University of South Florida, Tampa, Florida, United States of America; 2 Department of Biochemistry and Molecular Genetics, University of Illinois at Chicago, Chicago, Illinois, United States of America; 3 Burn and Shock Trauma Research Institute, Department of Surgery, Loyola University Chicago Stritch School of Medicine, Maywood, IL, United States of America; 4 Department of Biochemistry, Medical College of Wisconsin, Milwaukee, Wisconsin, United States of America; Medical College of Georgia, Augusta, UNITED STATES

## Abstract

We observed in PRESTO-Tango β-arrestin recruitment assays that the α_1_-adrenergic receptor (AR) antagonist prazosin activates chemokine (C-X-C motif) receptor (CXCR)4. This prompted us to further examine this unexpected pharmacological behavior. We screened a panel of 14 α_1/2_- and β_1/2/3_-AR antagonists for CXCR4 and atypical chemokine receptor (ACKR)3 agonist activity in PRESTO-Tango assays against the cognate agonist CXCL12. We observed that multiple α_1_-AR antagonists activate CXCR4 (CXCL12 = prazosin = cyclazosin > doxazosin) and ACKR3 (CXCL12 = prazosin = cyclazosin > alfuzosin = doxazosin = phentolamine > terazosin = silodosin = tamsulosin). The two strongest CXCR4/ACKR3 activators, prazosin and cyclazosin, were selected for a more detailed evaluation. We found that the drugs dose-dependently activate both receptors in β-arrestin recruitment assays, stimulate ERK1/2 phosphorylation in HEK293 cells overexpressing each receptor, and that their effects on CXCR4 could be inhibited with AMD3100. Both α_1_-AR antagonists induced significant chemical shift changes in the ^1^H-^13^C-heteronuclear single quantum correlation spectrum of CXCR4 and ACKR3 in membranes, suggesting receptor binding. Furthermore, prazosin and cyclazosin induced internalization of endogenous CXCR4/ACKR3 in human vascular smooth muscle cells (hVSMC). While these drugs did not in induce chemotaxis in hVSMC, they inhibited CXCL12-induced chemotaxis with high efficacy and potency (IC_50_: prazosin—4.5 nM, cyclazosin 11.6 pM). Our findings reveal unexpected pharmacological properties of prazosin, cyclazosin, and likely other α_1_-AR antagonists. The results of the present study imply that prazosin and cyclazosin are biased or partial CXCR4/ACKR3 agonists, which function as potent CXCL12 antagonists. Our findings could provide a mechanistic basis for previously observed anti-cancer properties of α_1_-AR antagonists and support the concept that prazosin could be re-purposed for the treatment of disease processes in which CXCR4 and ACKR3 are thought to play significant pathophysiological roles, such as cancer metastases or various autoimmune pathologies.

## Introduction

α_1_-Adrenergic receptor (AR) antagonists are widely used as antihypertensive drugs, for the treatment of benign prostate hyperplasia, and off-label for the treatment of Raynaud’s syndrome[1–3]. Moreover, the α_1_-AR antagonist prazosin has recently been evaluated in clinical trials in patients with post-traumatic stress disorders and nightmares[4]. Evidence suggests that various α_1_-AR antagonists have *in vitro* cytotoxic activity in prostate and other cancer cell lines, and anti-proliferative and metastasis reducing effects in prostate cancer mouse models[2, 5]. While the exact molecular mechanisms underlying anti-cancer effects of α_1_-AR antagonists remain to be determined, they appear independent of the presence α_1_-ARs[2, 6].

Recently, we showed that α_1_-ARs form hetero-oligomeric complexes with chemokine (C-X-C motif) receptor (CXCR) 4 and atypical chemokine receptor (ACKR) 3 in human vascular smooth muscle cells (hVSMC), through which the chemokine receptors regulate α_1_-AR signaling and function[7–9]. Subsequently, we provided evidence for asymmetrical cross-regulation of CXCR4-mediated signaling and function by α_1_-ARs within the heteromeric receptor complex[10]. In these studies, we utilized PRESTO-Tango (parallel receptorome expression and screening via transcriptional output, with transcriptional activation following arrestin translocation[11]) assays to demonstrate that activation of the α_1b_-AR:CXCR4 heteromer with phenylephrine leads to cross-recruitment of β-arrestin to CXCR4, which could be inhibited with the α_1_-AR antagonist phentolamine[10]. During these studies, we also employed other α_1_-AR antagonists in pilot experiments and observed that prazosin induced β-arrestin recruitment to CXCR4 in the absence of α_1b_-AR, suggesting that prazosin may activate CXCR4. This observation prompted us to further examine this unexpected pharmacological behavior of an AR antagonist. Thus, we screened a panel of α_1/2_-AR and β_1/2/3_-AR antagonists for CXCR4 and ACKR3 agonist activity in PRESTO-Tango assays against CXCL12 (stromal cell-derived factor 1α), the cognate agonist of both receptors, and then further evaluated the pharmacological properties of the two strongest activators of CXCR4 and ACKR3 in recombinant and native cell systems. We observed that multiple α_1_-AR antagonists activated CXCR4 and ACKR3. Furthermore, we provide functional and structural evidence suggesting that prazosin and the related α_1_-AR antagonist cyclazosin are partial or biased agonists of CXCR4 and ACKR3, and that both drugs inhibit CXCL12-induced chemotaxis with high potency and efficacy. Our findings demonstrate unexpected pharmacological properties of α_1_-AR antagonists.

## Materials and methods

### Reagents

AMD3100 and all AR antagonists, except silodosin (Cayman Chemical) and terazosin (Santa Cruz Biotech), were purchased from Sigma-Aldrich. CXCL12 was from Protein Foundry.

### Cells

HEK293 cells were cultured in high-glucose Dulbecco’s Modified Eagle's Medium containing 1 mM sodium pyruvate, 2 mM L-glutamine, 10% FBS, 100 U/mL penicillin, and 100 μg/mL streptomycin. The HTLA cell line, a HEK293 cell line stably expressing a tTA-dependent luciferase reporter and a β-arrestin2-TEV fusion gene [11], was generously provided by the laboratory of Dr. Bryan Roth and maintained in high glucose Dulbecco’s Modified Eagle’s Medium supplemented with 10% FBS, 100 U/mL penicillin, 100 μg/mL streptomycin, 100 μg/mL hygromycin B, and 2 μg/mL puromycin. Human primary aortic smooth muscle cells (hVSMCs PCS-100-012) were obtained from ATCC. hVSMCs were cultured using vascular basal cell media (PCS-100-030, ATCC) with the addition of supplemental growth factors (hVSMCs PCS-100-042, PPAE PCS-100-041 (ATCC)) and 100 U/mL penicillin, 100 μg/mL streptomycin, and utilized within passages 2–5. All cells were cultured in a humidified environment at 37°C, 5% CO_2_.

### Plasmids and transfections

TANGO plasmids (CXCR4-TANGO, #66262; ACKR3-TANGO #66265) were from Addgene deposited by the laboratory of Dr. Bryan Roth. HA-tagged CXCR4 or ACKR3 were generated by PCR amplification using corresponding TANGO plasmids as cDNAs with primers carrying Xho I and Xba I sites and inserted in pcDNA3 with an N-terminal HA tag. All plasmids were verified by sequencing. HA-CXCR4 or HA-ACKR3 were transfected in HEK293 cells, while TANGO plasmids were transfected in HTLA cells, using Lipofectamine 3000 (Thermo Scientific) as per manufacturer’s protocol.

### PRESTO-TANGO β-arrestin recruitment assay

The assay was performed as recently described [7, 11–13]. HTLA cells (2.5x10^5^/well) were seeded in a 6-well plate and transfected with 750 ng of each of the TANGO plasmids using Lipofectamine 3000 (ThermoScientific). The following day, transfected HTLA cells (75,000 cells/well) were plated onto Poly-L-Lysine pre-coated 96-well microplates and allowed to attach to the plate surface for at least 4 hours prior to treatment. Cells were treated with receptor ligands for 2h, ligands were replaced with fresh full medium and incubated overnight at 37°C, 5% CO_2_ in a humidified environment. To test the effects of AMD3100 (10 μM), cells were pre-incubated with AMD3100 for 15 min at 37°C before adding ligands. The following morning, medium was removed from cell culture plates and replaced with a 100 μL 1:5 mixture of Bright-Glo (Promega) and 1x HBSS, 20 mM HEPES solution. Plates were then incubated at room temperature for 20 min before measuring luminescence on a Biotek Synergy II plate reader.

### Western blotting

HEK293 cells were transfected in 12-well plates with 0.5 μg/well of DNA expressing either HA-CXCR4 or HA-ACKR3 using Lipofectamine 3000. Forty hours after transfection, cells were incubated with 100 nM of CXCL12, or 100 μ μM of prazosin or cyclazosin for various times as indicated in figure legends. To test the effects of AMD3100 (10 μM), cells were pre-incubated for 15 min at 37°C before adding 100 μM of prazosin or cyclazosin for 20 min. Cells were lysed with SDS lysis buffer and phospho-ERK1/2 and total ERK1/2 were examined with Western blotting with antibodies against phospho-ERK1/2 (Thr202/Tyr204) or total ERK1/2 (Cell Signaling #4370 and #4696).

### Receptor internalization assays

Assessment of receptor internalization upon drug treatment was achieved via flow cytometry. hVSMCs were incubated with 100 μM cyclazosin or prazosin at 37°C for 15 or 30 min. Cells were then blocked with 2% FBS in cold PBS for 30 min, followed by incubation with anti-CXCR4 (ACR-014, Alomone Labs) and anti-ACKR3 (MAB42273, R&D Systems) antibodies for 1h on ice. After washing two times, cells were incubated with secondary antibodies (Alexa 488-conjugated anti-mouse and Alexa 647-conjugated anti-rabbit) for 30 min on ice. Cells were counted on a BD FACS Canto II (BD Biosciences) flow cytometer. The geometric fluorescence intensities of at least 10^4^ cells were recorded and analyzed using the FlowJo software (Tree Star).

### Chemotaxis assays

Cell migration was assessed using the ChemoTx 96-well cell migration system, as described [8, 14]. The chemotactic index (CI) was calculated as the ratio of cells that transmigrated through the filter in the presence versus the absence (= PBS/control) of the test solutions.

### Cell viability assays

To assess the effects of AR antagonists on cell viability, hVSMC were treated with the drugs for 3 hours at 37°C, 5% CO_2_. Cells were then washed once with PBS, stained with Trypan Blue (0.4% 1:1 dilution), and manually counted with a hemocytometer. Cell viability and chemotaxis experiments were performed in parallel.

### Reductive methylation of membrane preparations

ChemiSCREEN Chem-1 membrane preparations for recombinant human CXCR4 and ACKR3 were purchased from EMD Millipore. Reductive methylation of the membrane preparations was performed as described previously [9, 15]

### Heteronuclear single quantum coherence (HSQC) NMR

Samples (200 μl) contained 50% of membrane preparations, 10% D2O, 2.5% DMSO-d6. Prazosin, cyclazosin and atipamezol were added at a final concentration of 200 μM. ^1^H-^13^C HSQC NMR experiments were carried out on a 900-MHz Bruker Avance Spectrometer equipped with a cryogenic probe. Data were processed and analyzed using the NMRPipe/NMRDraw software [16].

### Data analyses

Data are expressed as mean ± SE. Data were analyzed with the Mann Whitney U test, Kruskal-Wallis test with Dunn's multiple comparisons test or with non-linear regression analysis using the GraphPad-Prism-7 software. A 2-tailed p<0.05 was considered significant.

## Results and discussion

### Effects of AR antagonists on β-arrestin recruitment to CXCR4 and ACKR3

**Fig 1:**
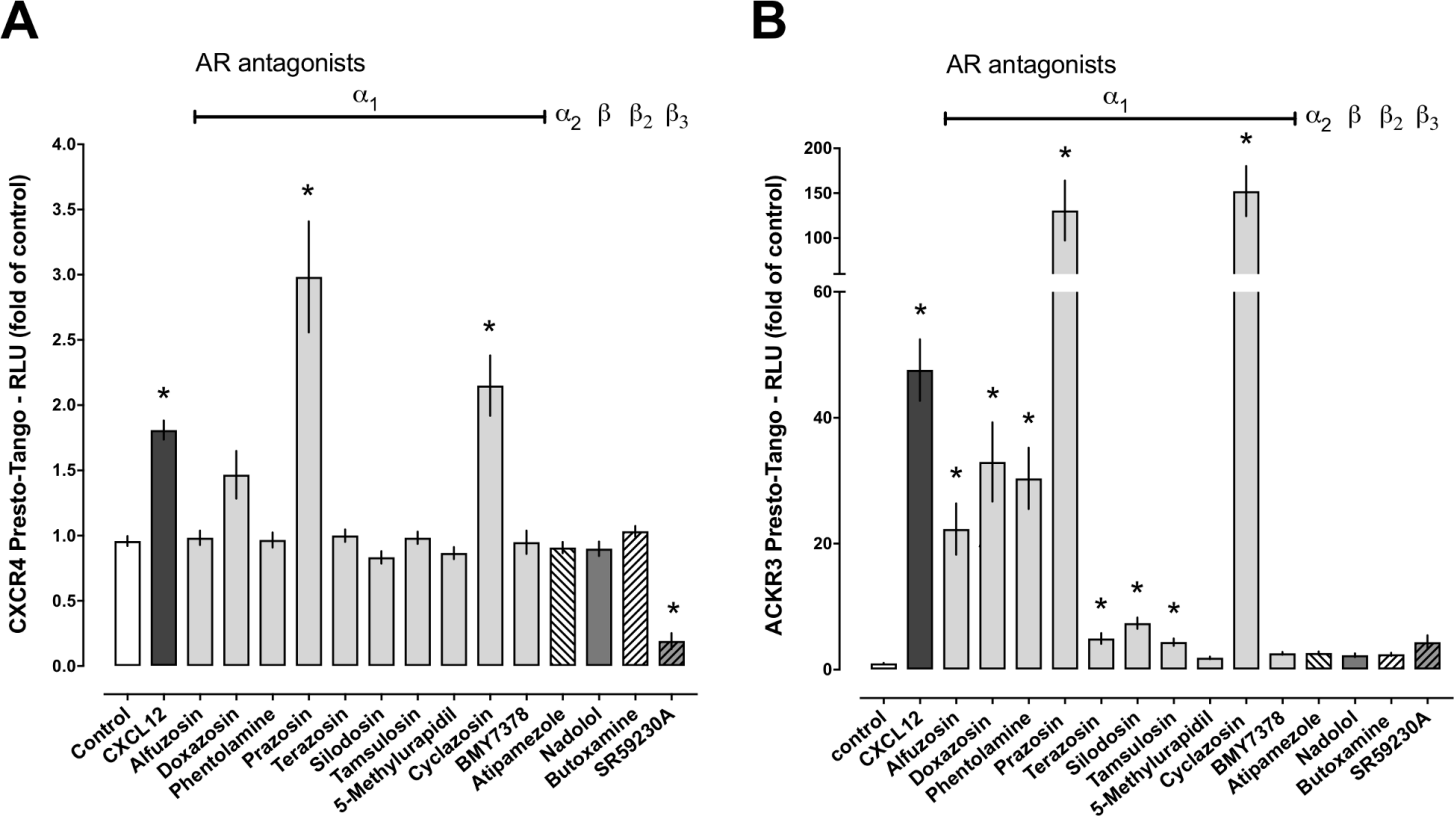
Screening of adrenergic receptor antagonists for CXCR4 and ACKR3 agonist activity in PRESTO-Tango β-arrestin recruitment assays. Data are mean ± SE from n = 4 independent experiments (in triplicates). Cells were stimulated with vehicle, 100 μM of individual AR antagonists or with 200 nM of CXCL12. Luminescence signals are expressed as fold of vehicle-treated cells (control, = 1). *: p<0.05 vs. vehicle (unstimulated). **A.** CXCR4 PRESTO-Tango assays. **B.** ACKR3 PRESTO-Tango assays.

We first tested a total of 14 AR antagonists at a concentration of 100 μM in PRESTO-Tangoβ-arrestin recruitment assays for CXCR4 and ACKR3 (Fig 1A and 1B). CXCL12, the natural agonist of both receptors, was used as a positive control and employed at a saturating concentration (200 nM), which is more than 40-*times* the EC_50_ concentration for CXCL12 in this assay system [10, 13, 17]. Consistent with our previous findings, the luminescence signal increased 1.8-*fold* upon activation of CXCR4 with CXCL12 (p<0.05 vs. unstimulated cells, Fig 1A)[10, 13, 17]. The CXCR4 PRESTO-Tango luminescence signals after stimulation with prazosin and the prazosin-related α_1_-AR antagonist cyclazosin increased 3.0-*fold* and 2.15-*fold*, respectively (p<0.05 vs unstimulated cells and p>0.05 vs. CXCL12 for both), suggesting that both drugs induce β-arrestin recruitment to CXCR4 with an efficacy comparable to CXCL12. Although the increase in luminescence signals for doxazosin did not reach statistical significance when the entire drug panel was compared with vehicle, doxazosin increased luminescence signals in PRESTO-Tango assays for CXCR4 1.5-*fold*, which was not significantly different from the luminescence signals induced by CXCL12 stimulation. While the β_3_-AR antagonist SR59230A reduced the luminescence signals of unstimulated cells, all other AR antagonists did not significantly affect luminescence signals (Fig 1A). The observation that SR59230A reduced baseline luminescence signals in the CXCR4 PRESTO-Tango assay could point towards inverse agonist activity of this drug, which remains to be determined. We then tested the panel of AR antagonists for ACKR3 agonist activity. As compared with the PRESTO-Tango assay for CXCR4, the luminescence signals in unstimulated cells were much lower in PRESTO-Tango assays for ACKR3, and CXCL12 stimulation induced a 48-*fold* increase of the signal (Fig 1B). We observed that several α_1_-AR antagonists also activated ACKR3 in PRESTO-Tango assays in the following rank order of potencies: CXCL12 = prazosin = cyclazosin > alfuzosin = doxazosin = phentolamine > terazosin = silodosin = tamsulosin.

**Fig 2:**
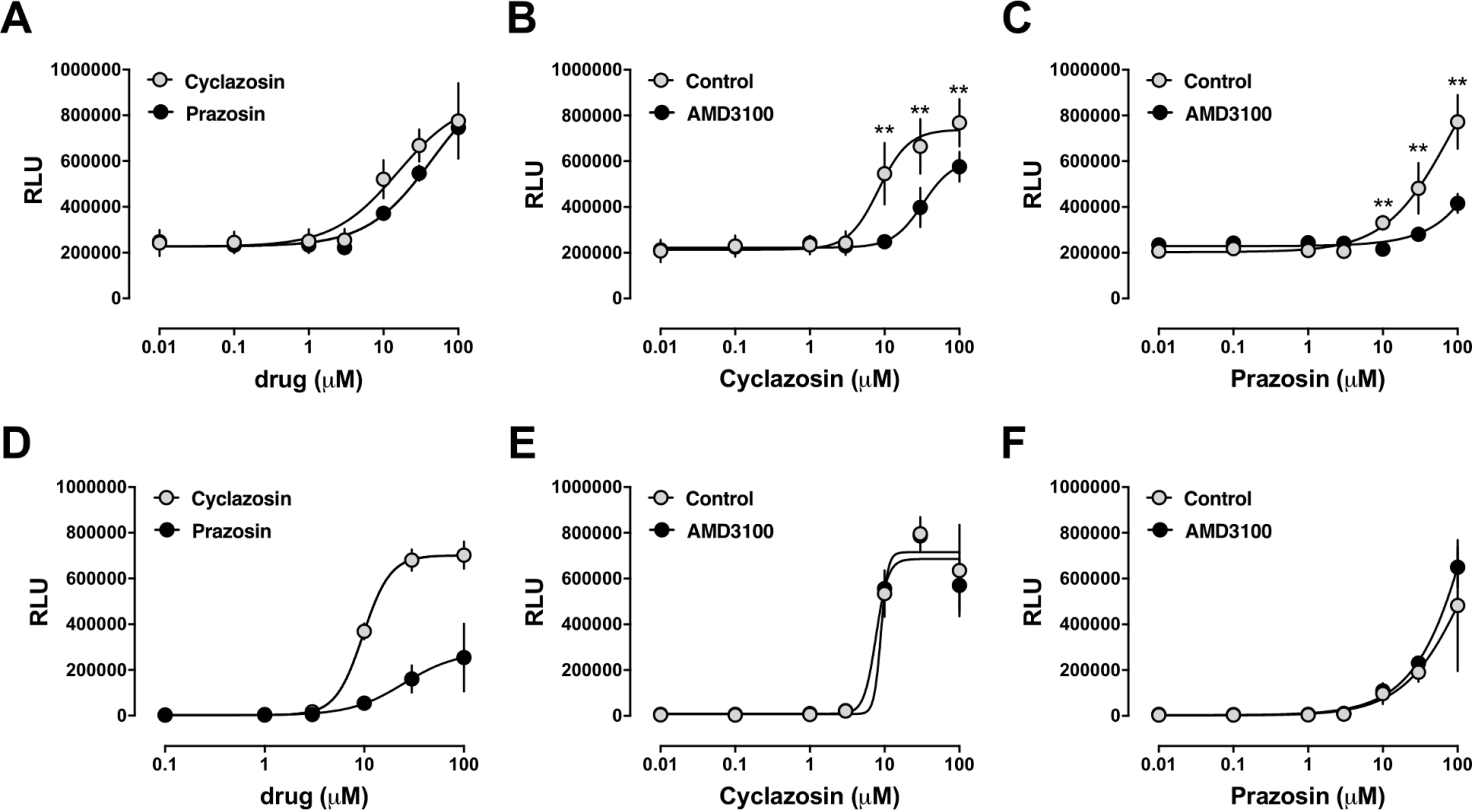
Dose-dependent effects of prazosin and cyclazosin in PRESTO-Tango β-arrestin recruitment assays for CXCR4 and ACKR3. Data are mean ± SE from n = 3 independent experiments (in triplicates). RLU: relative luminescence units. **A-C:** CXCR4 PRESTO-Tango assays. **A.** Grey circles: cells treated with cyclazosin. Black circles: cells treated with prazosin. **B.** Cells were pre-incubated with vehicle (control, grey circles) or AMD3100 (10 μM; black circles) for 15 min, followed by stimulation with cyclazosin. **: p<0.01 vs. cells pre-treated with AMD3100. **C.** Cells were pre-incubated with vehicle (control, grey circles) or AMD3100 (10 μM; black circles) for 15 min, followed by stimulation with prazosin. **: p<0.01 vs. cells pre-treated with AMD3100. **D-F:** ACKR3 PRESTO-Tango assays. **D.** Grey circles: cells treated with cyclazosin. Black circles: cells treated with prazosin. **E.** Cells were pre-incubated with vehicle (control, grey circles) or AMD3100 (10 μM; black circles) for 15 min, followed by stimulation with cyclazosin. **F.** Cells were pre-incubated with vehicle (control, grey circles) or AMD3100 (10 μM; black circles) for 15 min, followed by stimulation with prazosin.

We selected prazosin and cyclazosin as the strongest activators of the chemokine receptors to further characterize their pharmacological behavior. Next, we determined their dose-response characteristics in PRESTO-Tango assays and tested whether their effects can be blocked with the CXCR4 antagonist AMD3100. As shown in Fig 2A–2C, both drugs dose-dependently activated CXCR4 in PRESTO-Tango assays. The EC_50_ for prazosin was 45 ± 10 μM, and 16 ± 4 μM for cyclazosin. The effects of both drugs could be antagonized with AMD3100 (Fig 2B and 2C). Similarly, prazosin and cyclazosin activated ACKR3 in a dose-dependent manner (Fig 2D–2F; EC_50_: prazosin– 25 ± 7 μM, cyclazosin– 10 ± 0.6 μM). As expected, AMD3100 did not affect their activities in PRESTO-Tango assays for ACKR3 (Fig 2E and 2F).

### Prazosin and cyclazosin induce extracellular signal-regulated kinases 1/2 (ERK1/2) phosphorylation

**Fig 3:**
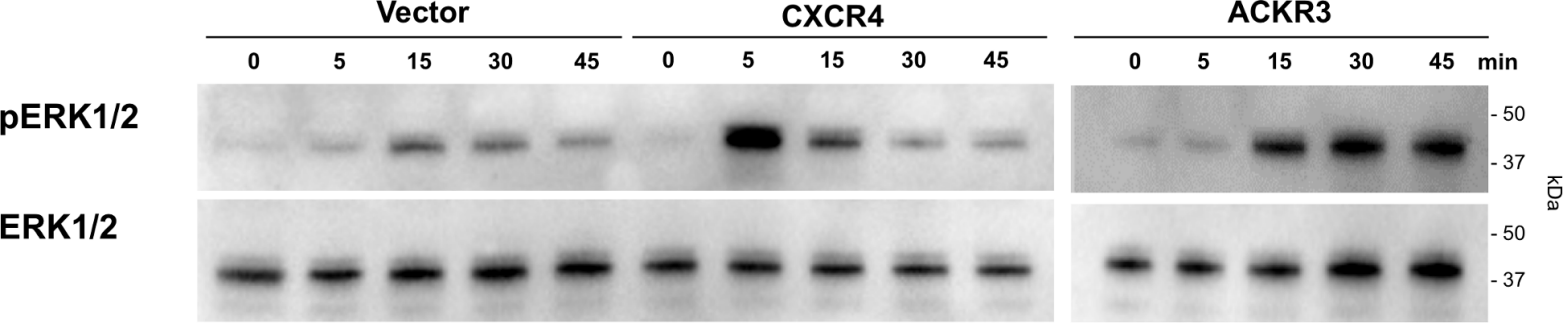
CXCL12-inudced ERK1/2 phosphorylation in HEK293 cells is augmented after transfection with CXCR4 and ACKR3. HEK293 cells were transfected with empty vector (left), CXCR4 (center) or ACKR3 (right) and stimulated with 100 nM of CXCL12 for various time periods as indicated. ERK1/2 phosphorylation was monitored by Western blotting of cell lysates with anti-phophoERK1/2 (pERK) and anti-ERK1/2. The migration position of molecular mass standards is indicated.

To determine whether prazosin and cyclazosin also activate other signaling events mediated by CXCR4 and ACKR3, we studied ERK1/2 phosphorylation in HEK293 cells. Consistent with the low expression of CXCR4 and ACKR3 in HEK293 cells [18–20], CXCL12-induced ERK1/2 phosphorylation could be augmented when cells were transfected with CXCR4 or ACKR3 (Fig 3). Thus, we utilized HEK293 cells transfected with CXCR4 or ACKR3 as an optimized test system.

**Fig 4:**
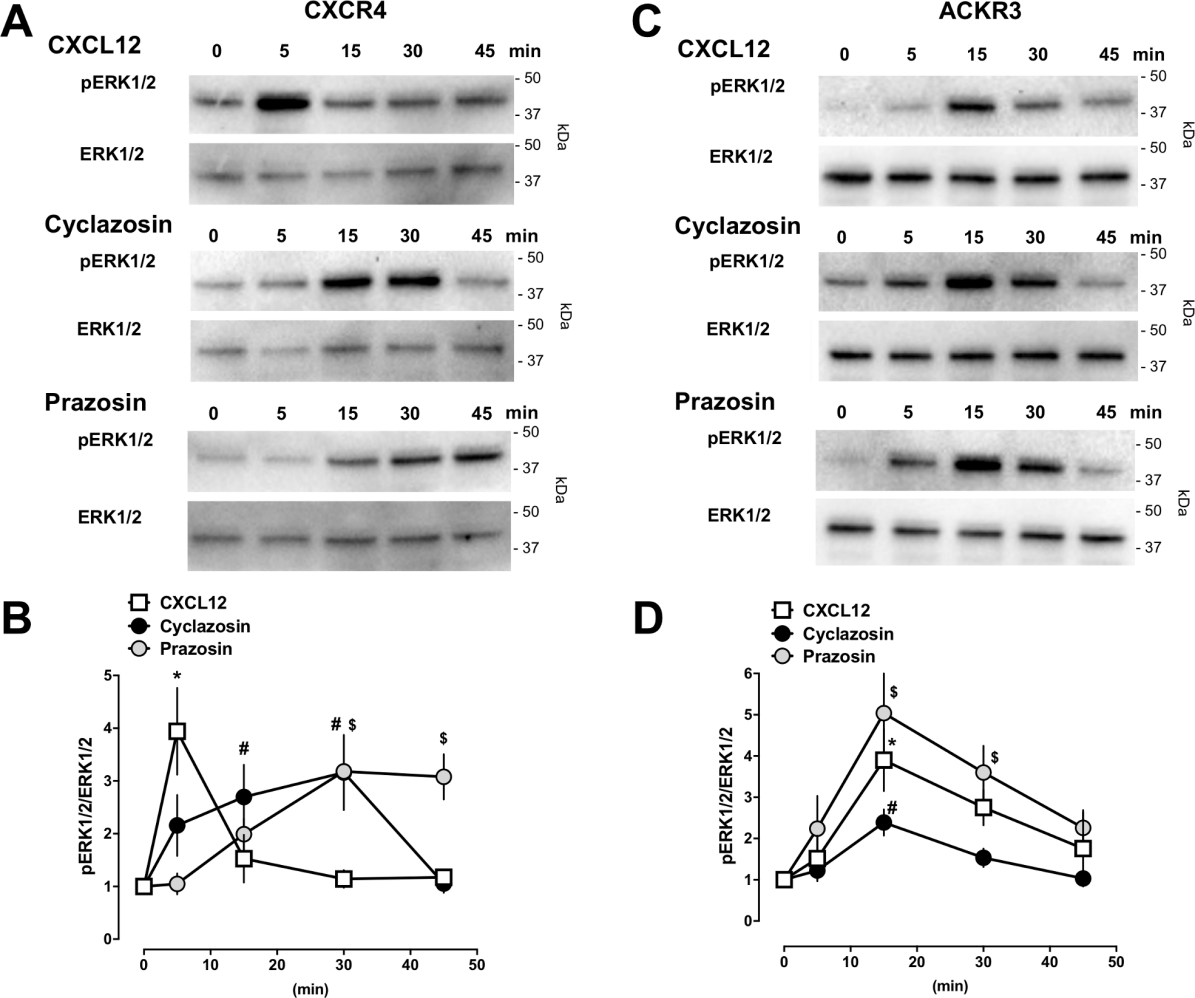
Prazosin and cyclazosin induce ERK1/2 phosphorylation. HEK293 cells transfected with CXCR4 (**A/B**) or ACKR3 (**C/D**) were stimulated with CXCL12 (100 nM), prazosin (100 μM) or cyclazosin (100 μM) for various time periods as indicated. ERK1/2 phosphorylation was monitored by Western blotting of cell lysates with anti-phophoERK1/2 (pERK) and anti-ERK1/2 (ERK1/2). **A.** Representative images from Western blot experiments with cells transfected with CXCR4. The migration position of molecular mass standards is indicated. **B.** Densitometric quantification of the band intensities, expressed as pERK1/2/ERK1/2, from n = 3 independent experiments as in A. Data are mean ± SE. Symbols (*: CXCL12; #: cyclazosin; $: prazosin) indicate significant differences vs. t = 0 min. **C.** Representative images from Western blot experiments with cells transfected with ACKR3. The migration position of molecular mass standards is indicated. **D.** Densitometric quantification of the band intensities, expressed as pERK1/2/ERK1/2, from n = 5 independent experiments as in B. Data are mean ± SE. Symbols (*: CXCL12; #: cyclazosin; $: prazosin) indicate significant differences vs. t = 0 min.

Fig 4A and 4C show representative images from Western blot experiments with cell lysates from CXCR4 (Fig 4A) and ACKR3 (Fig 4C) overexpressing cells and Fig 4B and 4D the densitometric quantifications of the band intensities from three independent experiments. Activation of cells overexpressing CXCR4 with CXCL12 caused a rapid and transient increase in ERK1/2 phosphorylation. When cells were stimulated with prazosin and cyclazosin, the degree of ERK1/2 phosphorylation was very similar to CXCL12 (4-*fold* increase with CXCL12 vs. 3-*fold* increase with prazosin and cyclazosin, Fig 4). While the time progression of ERK1/2 phosphorylation was delayed after stimulation with both drugs, ERK1/2 phosphorylation occurred over prolonged time periods, as compared to cells stimulated with CXCL12 (Fig 4B). The time progression of ERK1/2 phosphorylation in cells overexpressing ACKR3 was identical for stimulation with CXCL12, prazosin and cyclazosin (Fig 4C and 4D). While the degree of ERK1/2 phosphorylation was similar upon stimulation with CXCL12 and prazosin, the effects of cyclazosin appeared to be weaker.

**Fig 5:**
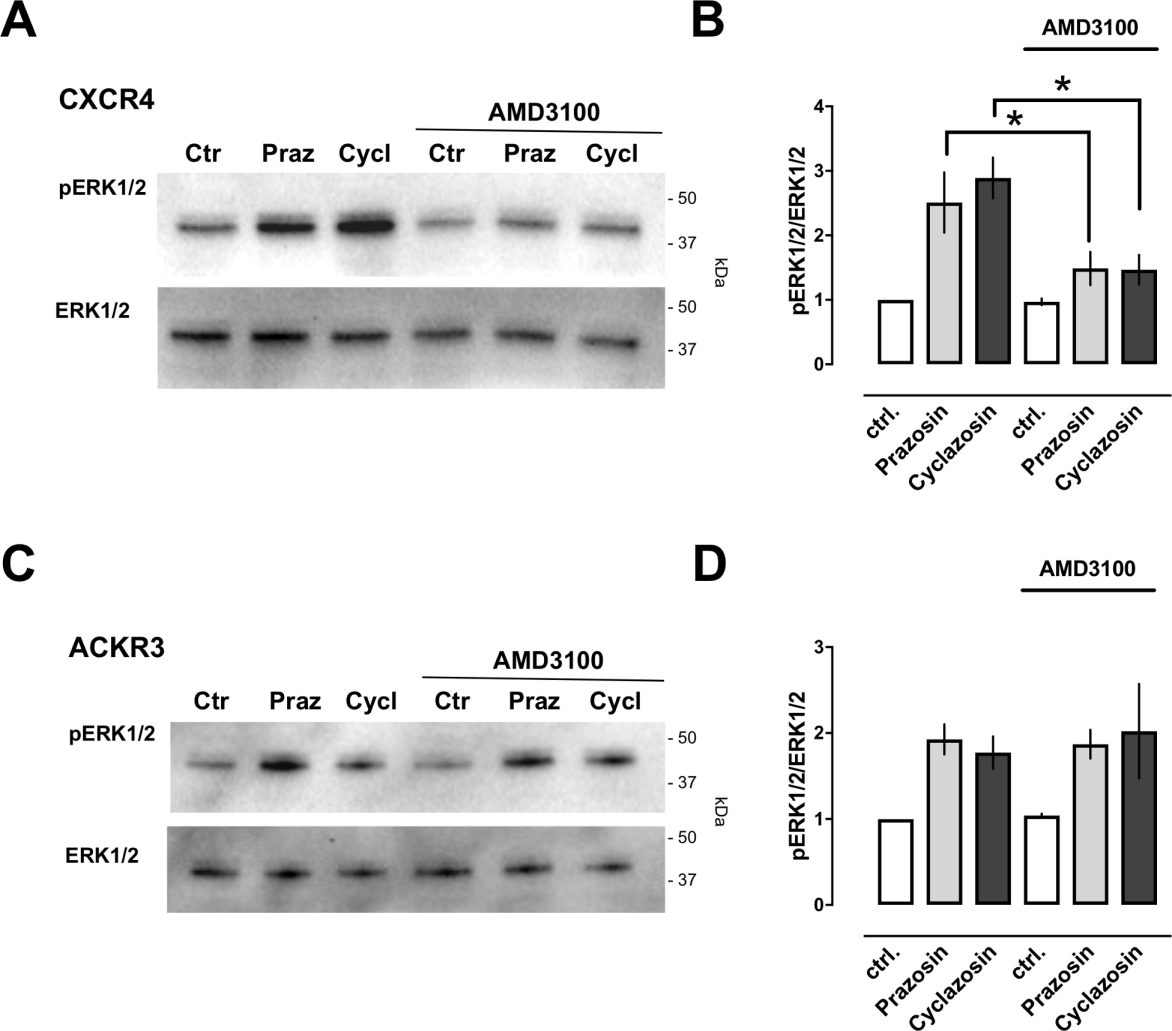
Effects of AMD3100 on prazosin and cyclazosin induced ERK1/2 phosphorylation. ERK1/2 phosphorylation was measured and analyzed as in Fig 3. Cells were pre- incubated with AMD3100 (10 μM, 15 min), followed by stimulation with vehicle (control, ctrl), prazosin (Praz) or cyclazosin (Cycl, 100 μM each) for 20 min. **A.** Representative images from Western blot experiments with cells transfected with CXCR4. The migration position of molecular mass standards is indicated. **B.** Densitometric quantification of the band intensities, expressed as pERK1/2/ERK1/2, from n = 5 independent experiments as in A. Data are mean ± SE. *: p<0.05. **C.** Representative images from Western blot experiments with cells transfected with ACKR3. The migration position of molecular mass standards is indicated. **D.** Densitometric quantification of the band intensities, expressed as pERK1/2/ERK1/2, from n = 5 independent experiments as in A. Data are mean ± SE.

As observed in PRESTO-Tango assays, prazosin- and cyclazosin-induced ERK1/2 phosphorylation in cells overexpressing CXCR4 could be antagonized with AMD3100 (Fig 5A and 5B), but not in cells overexpressing ACKR3 (Fig 5C and 5D). It has been shown previously that G protein-mediated ERK1/2 phosphorylation occurs rapidly and transient, whereas β-arrestin-mediated ERK1/2 phosphorylation occurs more slowly and over prolonged time periods[21]. While CXCR4 activation with CXCL12 induces G protein- and β-arrestin-mediated signaling, ACKR3 is thought not couple to G proteins but recruits β-arrestin to the receptor, leading to signaling upon agonist binding[22, 23]. Thus, the time progression and the duration of ERK1/2 phosphorylation induced by prazosin and cyclazosin are suggestive of activation of β-arrestin mediated signaling of CXCR4 and ACKR3, whereas CXCL12 appears to preferentially activate G protein-mediated signaling of CXCR4 in our test system.

### Prazosin and cyclazosin induce chemical shift changes in the ^1^H-^13^C heteronuclear single quantum correlation (HSQC) spectrum of CXCR4 and ACKR3 in membrane preparations

**Fig 6:**
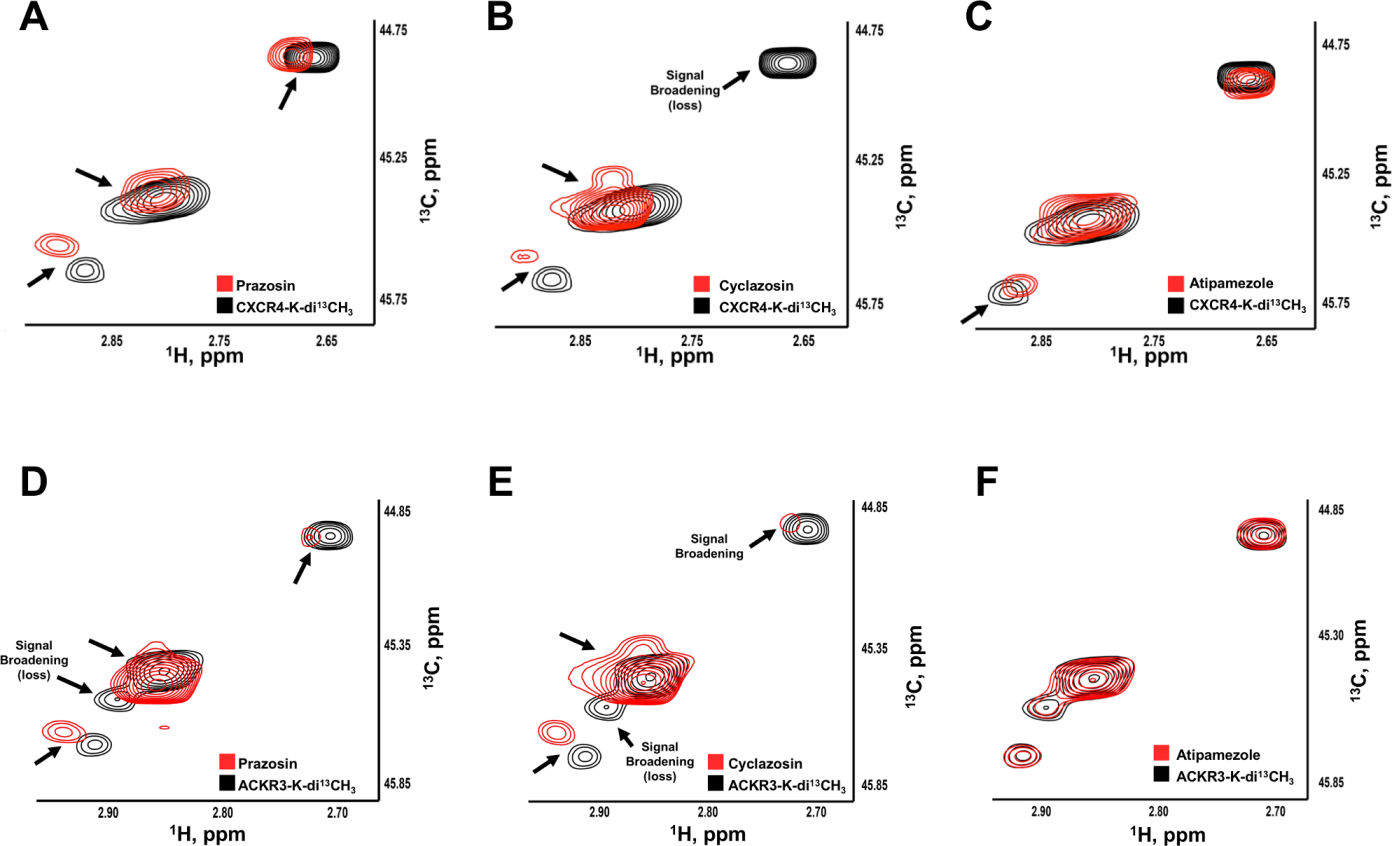
Prazosin and cyclazosin induce chemical shift changes in the NMR spectra of CXCR4 and ACKR3 in membranes. ^1^H-^13^C HSQC spectra of reductively methylated CXCR4 (**A-C**) and ACKR3 (**D-F**) membrane preparations were recorded without (black) and with (red) 200 μM prazosin (**A/D**), cyclazosin (**B/E**) or atipamezol (**C/F**). Black arrows indicate significant differences in chemical shifts or broadening (loss) of the signal.

Our observations on the signaling properties of prazosin and cyclazosin suggested that both drugs bind and activate CXCR4 and ACKR3. Thus, we sought to provide direct biophysical evidence for their binding to the receptors. We employed nuclear magnetic resonance (NMR) spectroscopy and utilized ^13^C-labeled methylated membranes prepared from cells overexpressing CXCR4 or ACKR3 to closely mimic native conditions for receptor folding and interactions with the plasma membrane. We have utilized this strategy previously to assess ligand binding to CXCR4 and α_1a_-AR [9, 13]. We selected atipamezole as a control drug that did not activate CXCR4 or ACKR3 in PRESTO-Tango assays. The overlaid ^1^H -^13^C- heteronuclear single quantum coherence (HSQC) spectra of CXCR4 and ACKR3 with and without 200 μM of the individual drugs are shown in Fig 6. Prazosin (Fig 6A and 6D) and cyclazosin (Fig 6B and 6E) induced significant line-broadening and/or chemical shift perturbations in the NMR spectra of CXCR4- and ACKR3-containing membranes, indicative of a global structural rearrangement of the receptor induced by drug binding. These large effects could not be detected upon addition of atipamezole (Fig 6C and 6F). The observations that all signals, including the ^13^C-methylated N-terminal amino group[15], were significantly perturbed by the addition of prazosin and cyclazosin suggest that both drugs affect the conformations of the receptors, thus providing biophysical evidence for prazosin and cyclazosin binding to CXCR4 and ACKR3 in membranes.

### Prazosin and cyclazosin induce internalization of CXCR4 and ACKR3 and inhibit CXCL12 induced chemotaxis in human vascular smooth muscle cells

**Fig 7:**
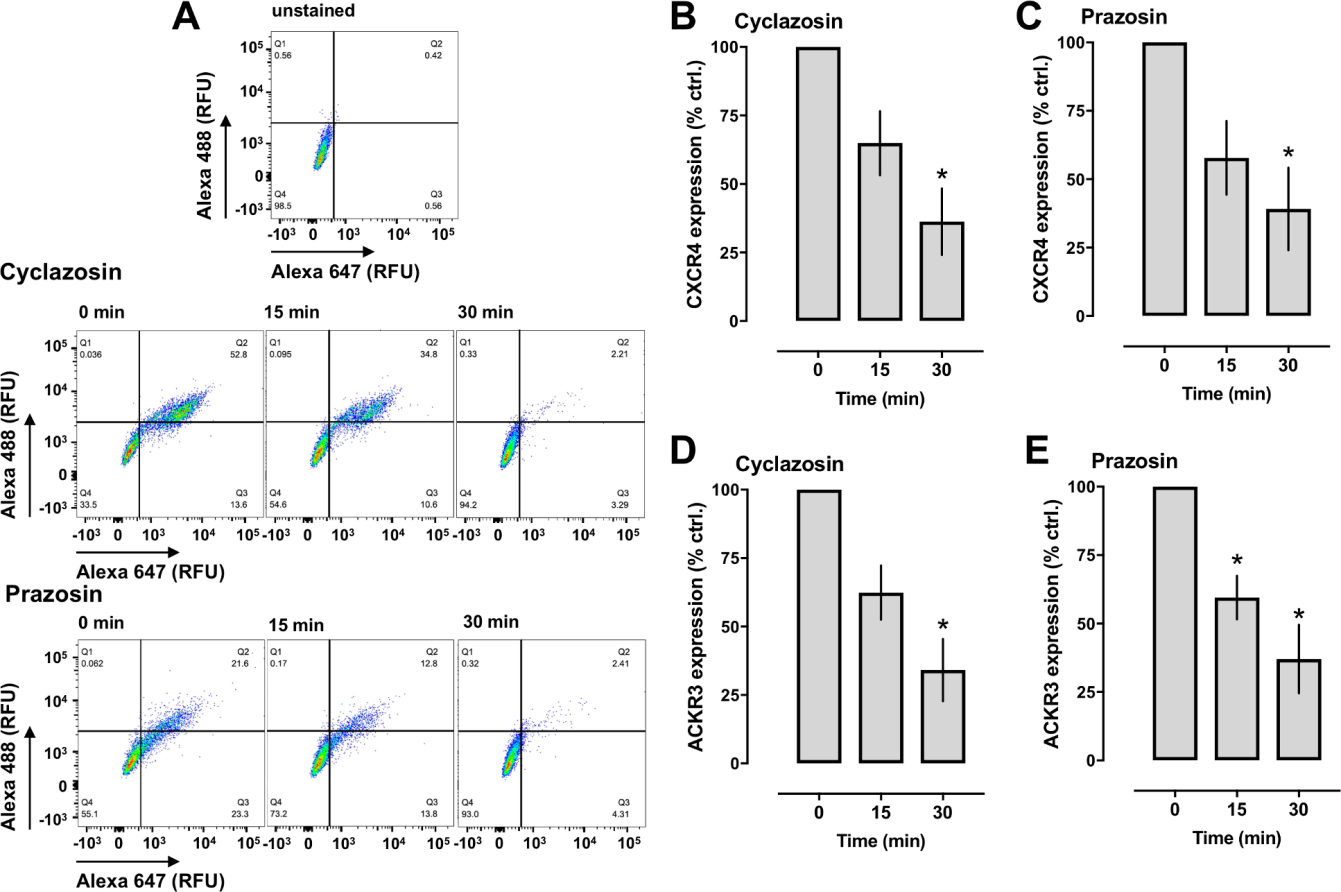
Prazosin and cyclazosin induce internalization of CXCR4 and ACKR3 in hVSMCs. **A.** hVSMC were treated with 100 μM cyclazosin or prazosin at 37°C for 15 or 30 min, stained with anti-CXCR4/Alexa 647-conjugated anti-rabbit and anti-ACKR3/Alexa 488-conjugated anti-mouse and analyzed for receptor expression via flow cytometry. RFU: relative fluorescence units. The horizontal and vertical lines show the gating thresholds for CXCR4 (Alexa 647) and ACKR3 (Alexa 488). **B-E**. Quantification of CXCR4 (**B/C**) and ACKR3 (**D/E**) cell surface expression by flow cytometry. Cells were stimulated with 100 μM cyclazosin (**B/D**) or prazosin (**C/E**) as indicated. Data are mean ± SE from 4 independent experiments. *: p<0.05 vs. t = 0 min.

Because β-arrestin recruitment upon agonist binding leads to internalization of CXCR4 and ACKR3, we tested in flow cytometry experiments whether both drugs reduce cell surface expression of endogenous CXCR4 and ACKR3 in hVSMCs. Fig 7A shows representative 2-dimensional scatter plots for the detection of both receptors over a 30 min time period after stimulation of hVSMC with prazosin or cyclazosin and Fig 7B–7E the quantification of receptor cell surface expression from four independent experiments. We observed that prazosin and cyclazosin reduced the expression of both receptors in a time-dependent manner. These findings are consistent with the observed effects of the drugs in recombinant test systems and imply that prazosin and cyclazosin bind to endogenous CXCR4 and ACKR3, leading to β-arrestin recruitment to the receptors and their subsequent internalization.

**Fig 8:**
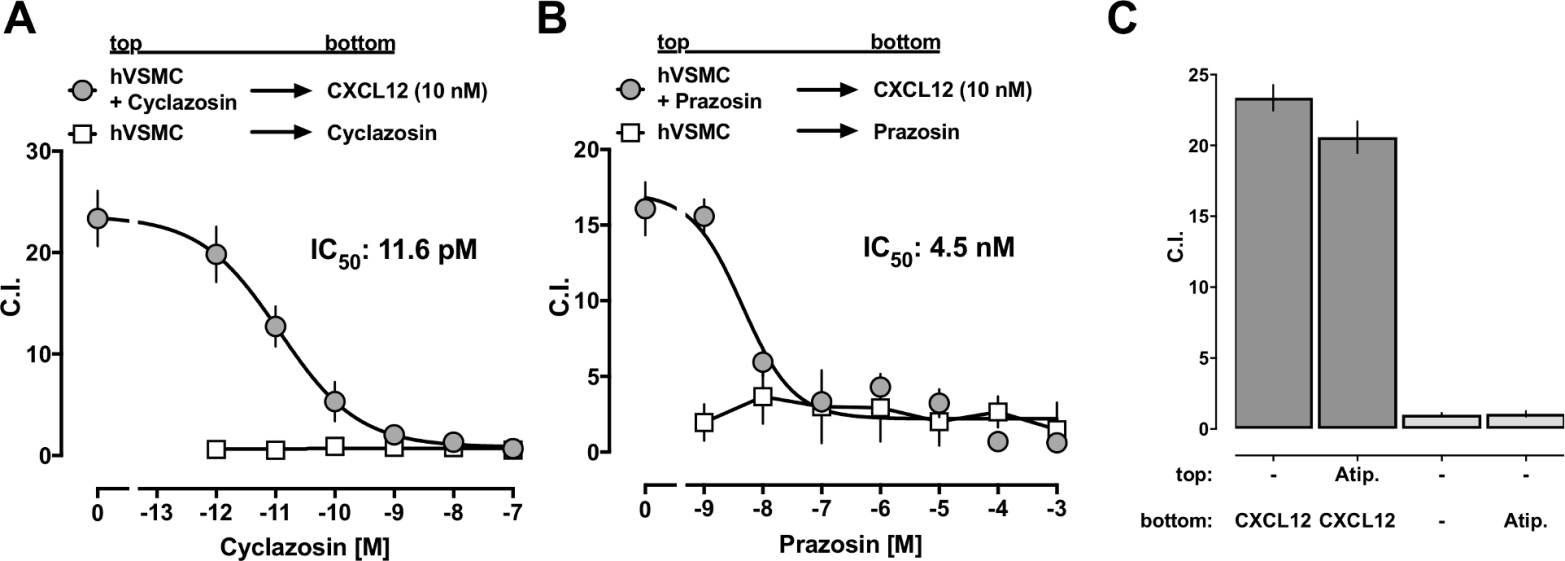
Prazosin and cyclazosin inhibit CXCL12-mediated chemotaxis of hVSMCs. **A.** Migration of hVSMCs towards cyclazosin (open squares) and of hVSMCs in the presence of cyclazosin towards 10 nM of CXCL12 (grey circles). Data are mean ± SE from 3 independent experiments. **B.** Migration of hVSMCs towards prazosin (open squares) and of hVSMCs in the presence of prazosin towards 10 nM of CXCL12 (grey circles). Data are mean ± SE from 3 independent experiments. **C.** Migration of hVSMCs in the presence of vehicle or atipamezole (Atip., 100 μM) towards CXCL12 (10 nM, dark grey bars) and towards vehicle or Atip. (100 μM, light grey bars). Data are mean ± SE from 3 independent experiments.

Next, we addressed whether prazosin and cyclazosin also modulate CXCR4- and ACKR3-mediated hVSMC function. Because VSMCs are known to migrate towards CXCR4 and ACKR3 agonists[10, 13, 23], we utilized chemotactic responses of hVSMCs as a functional read-out. Neither of the drugs, however, induced chemotaxis of hVSMCs (Fig 8A and 8B, open squares). As both drugs reduced cell surface expression of CXCR4 and ACKR3 in hVSMCs (Fig 7A–7E), we then tested whether prazosin and cyclazosin may attenuate migration of hVSMCs towards CXCL12. We detected that cyclazosin fully inhibited CXCL12-induced chemotaxis of hVSMCs in a dose-dependent manner. The IC_50_ for the inhibition of CXCL12-mediated chemotaxis by cyclazosin was 11.6 pM (Fig 8A). Similarly, prazosin fully inhibited CXCL12-induced chemotaxis with an IC_50_ of 4.5 nM (Fig 8B). In contrast, atipamezole did not affect CXCL12-induced chemotaxis (Fig 8C). To address the possibility that the inhibitory effects of prazosin and cyclazosin are caused by cytotoxicity in hVSMCs, we assessed viability of hVSMCs by Trypan Blue exclusion in parallel experiments under identical conditions. As compared with vehicle treated cells (95 ± 4% viability), exposure of hVSMCs to 100 μM atipamezole (96 ± 5% viability), 1 mM of prazosin (86 ± 8% viability) or 1 mM of cyclazosin (98 ± 8% viability) did not significantly reduce cell viability (p>0.05 vs. vehicle for all).

Collectively, our findings suggest that prazosin and cyclazosin bind CXCR4 and ACKR3 in membranes, lead to β-arrestin recruitment to the receptors, activate ERK1/2 phosphorylation and receptor internalization, and inhibit CXCL12-mediated chemotaxis with high potency and efficacy.

The pharmacological behavior of prazosin and cyclazosin that we observed in the present study is not without precedence. For example, TC14012 was initially described as a CXCR4 inhibitor and subsequently identified as an ACKR3 agonist that induces β-arrestin recruitment to the receptor[20]. Similarly, AMD3100 was shown to function as an allosteric ACKR3 agonist[24].

The observed discrepancy of the potencies of prazosin and cyclazosin to activate recombinant receptors in PRESTO-Tango assays (low μM range) and to inhibit CXCL12-induced chemotaxis in hVSMCs (low nM–pM range) is 1–2 orders of magnitude larger than previously reported discrepancies between functional and binding affinities of α_1_-AR antagonists when tested in recombinant and various endogenous systems[25]. Nevertheless, we observed a similar discrepancy in the potencies of phenylephrine to activate α_1b_-AR in PRESTO-Tango assays and to induce chemotaxis in hVSMCs previously[10]. Thus, the large differences in the potencies of prazosin and cyclazsosin in the present study could be explained by variations of their potencies for recombinant and endogenous receptors. Moreover, it appears possible that both drugs exert differential pharmacological behaviors upon binding to receptor homomers, which is likely in the expression system, and heteromers in hVSMCs[7–10]. We reported previously that phenylephrine stimulation induces β-arrestin cross-recruitment to and internalization of CXCR4 within the α_1b_-AR:CXCR4 heteromer, and that phenylephrine inhibits CXCL12-mediated chemotaxis of hVSMC with high potency and efficacy[10]. Although phenylephrine-induced β-arrestin cross-recruitment to CXCR4 could be prevented by phentolamine, a high dose of phentolamine also cross-inhibited CXCL12-mediated chemotaxis of hVSMCs[10]. We interpreted these findings to reflect asymmetrical cross-inhibition at the α_1B_-AR:CXCR4 heteromeric complex, a pharmacological behavior that has been reported for other G protein-coupled receptor heteromers[10, 26]. As phentolamine and multiple other α_1_-AR antagonists also activated β-arrestin recruitment to ACKR3 in the present study, it is likely that these dugs share the pharmacological behavior of prazosin and cyclazosin, and that direct interactions of phentolamine, prazosin and cylcazosin with ACKR3 and/or CXCR4 contribute to their inhibitory effects on CXCL12-induced chemotaxis of hVSMC.

Our findings that prazosin and cyclazosin induce chemical shift changes in the NMR spectra of the receptors in membranes, along with the observed time progression of the drug-induced ERK1/2 phosphorylation and their inability to induce chemotaxis in hVSMCs, show that both drugs lack full and balanced agonist activity, suggesting that they likely function as partial or biased agonists at CXCR4 and ACKR3. Irrespective of the precise underlying molecular mechanisms, the high potency and efficacy of prazosin and cyclazosin to inhibit CXCL12-mediated chemotaxis of native cells indicates that they exert these properties at pharmacologically relevant concentrations. While cyclazosin is not being used in patients, the pharmacokinetic profile of prazosin is well described. After oral standard doses of 2–5 mg prazosin, plasma concentrations reach 50–100 nM[27]. The IC_50_ concentration of prazosin to inhibit CXCL12-induced chemotaxis of 4.5 nM in the present study is well below clinically relevant plasma concentration in humans. This implies that standard doses of prazosin should antagonize CXCR4/ACKR3-mediated cell migration and inhibit receptor functions *in vivo*. Interestingly, several large database analyses already suggested that men treated with α_1_-AR antagonists may have a reduced incidence of prostate and bladder cancer [2, 28–31], in both of which CXCR4 and ACKR3 have been reported to contribute to the cancer pathogenesis[32–34]. The findings of the present study may provide a mechanistic basis for previously observed anti-cancer effects of α_1_-AR antagonists[2, 5]. In conclusion, the present study reveals unforeseen pharmacological properties of prazosin, cyclazosin and likely other α_1_-AR antagonists, which support the concept that prazosin could be re-purposed for the treatment of disease processes in which CXCR4 and ACKR3 are thought to play significant pathophysiological roles, such as cancer metastases or various autoimmune pathologies[35, 36].

## Supporting information

S1 Dataset All data sets are provided in this file. (XLSX)
